# SPOCK1 Promotes the Development of Hepatocellular Carcinoma

**DOI:** 10.3389/fonc.2022.819883

**Published:** 2022-02-03

**Authors:** Lóránd Váncza, Katalin Karászi, Bálint Péterfia, Lilla Turiák, Katalin Dezső, Anna Sebestyén, Andrea Reszegi, Gábor Petővári, András Kiss, Zsuzsanna Schaff, Kornélia Baghy, Ilona Kovalszky

**Affiliations:** ^1^ 1^st^ Department of Pathology and Experimental Cancer Research, Semmelweis University, Budapest, Hungary; ^2^ Faculty of Information Technology and Bionics, Pázmány Péter Catholic University, Budapest, Hungary; ^3^ MS Proteomics Research Group, Research Centre for Natural Sciences, Eötvös Loránd Research Network, Budapest, Hungary; ^4^ 2^nd^ Department of Pathology, Semmelweis University, Budapest, Hungary

**Keywords:** hepatocellular cancer (HCC), SPOCK1 in the liver, SPOCK1 and syndecan-1, SPOCK1 in mitochondrion, effect of SPOCK1 inhibition and overexpression

## Abstract

The extracellular matrix proteoglycan SPOCK1 is increasingly recognized as a contributor to the development and progression of cancers. Here, we study how SPOCK1, which is present in non-tumorous hepatocytes at low concentrations, promotes the development and progression of malignant hepatocellular tumors. Although SPOCK1 is an extracellular matrix proteoglycan, its concentration increases in the cytoplasm of hepatocytes starting with very low expression in the normal cells and then appearing in much higher quantities in cells of cirrhotic human liver and hepatocellular carcinoma. This observation is similar to that observed after diethylnitrosamine induction of mouse hepatocarcinogenesis. Furthermore, syndecan-1, the major proteoglycan of the liver, and SPOCK1 are in inverse correlation in the course of these events. In hepatoma cell lines, the cytoplasmic SPOCK1 colocalized with mitochondrial markers, such as MitoTracker and TOMM20, a characteristic protein of the outer membrane of the mitochondrion and could be detected in the cell nucleus. SPOCK1 downregulation of hepatoma cell lines by siRNA inhibited cell proliferation, upregulated p21 and p27, and interfered with pAkt and CDK4 expression. A tyrosine kinase array revealed that inhibition of SPOCK1 in the liver cancer cells altered MAPK signaling and downregulated several members of the Sarc family, all related to the aggressivity of the hepatoma cell lines. These studies support the idea that SPOCK1 enhancement in the liver is an active contributor to human and rodent hepatocarcinogenesis and cancer progression. However, its mitochondrial localization raises the possibility that it has a currently unidentified physiological function in normal hepatocytes.

## 1 Introduction

Several factors influence the contest between cancer and its prevention as well as treatment. Pollution of the environment, sedentary lifestyle, inappropriate food consumption, etc., go together with the inappropriate response of our body to injured molecules that gain oncogenic potentials. Among the factors, the current tumor management still fails to consider the importance of extracellular matrix (ECM) and stromal components of cancers, although they are known to be active participants in the development and progression of malignant tumors (1–3).

In the last 30 years, several publications proved the role of proteoglycans in the physiology and pathology of the liver. Although according to the literature syndecan-1 is the major heparan sulfate proteoglycan of the healthy liver, all four members of the family have been reported in increased amounts in various chronic liver diseases, such as liver fibrosis, cirrhosis (4, 5), cholestasis (6), non-alcoholic fatty liver disease (NAFLD) (7), and cancer (8, 9). Glypican-3 is a typical marker of hepatocellular carcinoma, detected not only in the tumor cells but also in the circulation (10). The fact that it is hardly expressed in the normal healthy liver makes glypican-3 a potential therapeutic target (11). In the healthy liver, agrin and perlecan are the components of basement membranes of the blood vessels; their increased amounts can be detected not only there but also in the connective tissue of the liver cirrhosis and cancer (12). The importance of agrin was suggested in the early phase of cancer development (11). In contrast with published data (13), versican, the only hyalectan ECM proteoglycan, is deposited mainly in the stroma of liver cancer according to the Human Protein Atlas.

SPOCK1/Testican-1 was discovered in 1992, isolated from the seminal plasma (14), and then cloned from brain tissue in 1997 (15). The limited information about the protein verifies its presence in the brain (16), the neuromuscular synapsis (17), the ECM, the endothelial cells, and evidently the testis. Through MT1-MMP, SPOCK1 inhibits MMP2 (18) and a couple of other proteases (19). It is involved in the mechanism of sepsis (20). Since the first study reported its oncogenic role in 2011, a number of accounts described the implication of SPOCK1 in the poor outcome in almost all types of cancers, from glioblastoma to prostate tumors (21–24). The mechanism of its action is still not completely understood; inhibition of apoptosis, upregulation of the Akt pathway, and involvement in epithelial–mesenchymal transition (EMT) are speculated, among others (23, 25–28). SPOCK1 is also implicated in the development and progression of liver cancer (29).

In spite of these reports, SPOCK1 is still considered as an ECM proteoglycan, and interestingly hardly any account paid attention that it can be detected in tumor cells of surgically removed human cancer tissues, and also in the cytoplasm of cancer cell lines (30). Human Protein Atlas provides several examples of cancer tissues expressing ample amounts of SPOCK1 in the tumor cells. The presence of SPOCK1 in epithelial tumors indicates that SPOCK1 likely can be found in resident non-tumorous epithelial cells, although in very low amounts. Based on this background, we investigated if a) healthy or diseased hepatocytes express SPOCK1 and b) SPOCK1 plays a mechanistic role during hepatocarcinogenesis and cancer progression. To this end, we performed a series of assays on an experimental hepatocarcinogenesis model, studied human liver cancers, and quantified how SPOCK1 silencing or upregulation influenced the aggressiveness of hepatoma cell lines.

## 2 Materials and Methods

### 2.1 Materials

If it is not indicated otherwise, all materials were purchased from Merck KGaA (Darmstadt, Germany). For the antibodies used see **Supplementary Table 1**.

### 2.2 Cell Culture

HepG2 (ATCC HB-8065) was obtained from American Type Culture Collection (ATCC, Manassas, VA, USA); HLE (JCRB0404) and Huh7 (JCRB0403) were obtained from the Japanese Collection of Research Bioresources Cell Bank. Cell lines were maintained in Dulbecco’s Modified Eagle Medium (DMEM) Low Glucose (D5546) supplemented with 10% fetal bovine serum (FBS; FB-1001B/500, Biosera, Kansas City, MO, USA), 2 mM of l-glutamine (XC-T1715/100; Biosera), 100 unit/ml of penicillin, and 100 μg/ml of streptomycin (P0781-100ML).

### 2.3 Double-Fluorescent Immunocytochemistry: SPOCK1 and TOMM20

Cells were plated on coverslips in 6-well plates (2 × 10^5^ cell/well) and cultivated in a complete growth medium for 24 h. Cells were washed three times with phosphate-buffered saline (PBS; containing 137 mM of NaCl, 2.7 mM of KCl, 10 mM of Na_2_HPO_4_, and 1.8 mM of KH_2_PO_4_, pH 7.5) and then fixed with ice-cold methanol for 10 min. Fixed cells were washed three times with PBS followed by blocking with 5% w/v bovine serum albumin (BSA) in PBS. SPOCK1 and TOMM20 primary antibodies were applied overnight in 1% w/v of BSA at 4^○^C. Next day, the coverslips were washed three times with PBS and incubated with fluorescent-labeled Alexa Fluor 488 and Alexa Fluor 568-conjugated secondary antibodies in 1% w/v of BSA containing DAPI (1:200; D9542) for 1 h at room temperature. The cells were washed and mounted using Fluoromount (F4680; Merck KGaA). In the case of the negative controls, the primary antibodies were omitted.

### 2.4 SPOCK1 Silencing in HLE and Huh7 Cell Lines

The transfection was performed using Lipofectamine™ 3000 Transfection Reagent (L3000008, Life Technologies, Carlsbad, CA, USA) and ON-TARGETplus Human SPOCK1 siRNA (L-013724-01-0005, Dharmacon Inc., Lafayette, CO, USA). The target sequences of the SPOCK1 siRNA were as follows: “CCUACAAAGAACAUCGUAA”, “GGGUUGGACCUUCGAAUUU”, “CGAUGGAGCCACAUUAAUA”, and “GGUGUAAUGAGGAGGGCUA”. To perform the transfection, cells were seeded in 6-well plates at a density of 1.5 × 10^5^ cell/well from Huh7 and 1 × 10^5^ cell/well from HLE and cultured for 24 h in a complete growth medium. In each well, 6 µl of transfection reagent and 50 pmol of SPOCK1 siRNA or 50 pmol of scrambled siRNA as negative control were used. Transfection efficacy was controlled with GAPDH siRNA. Twenty-four hours after the transfection, the medium was changed to a fresh growth medium. Forty-eight hours after transfection, the cells and conditioned medium were collected for follow-up experiments.

### 2.5 Expression Plasmids and Transfection of HepG2 Cells

The expression plasmid for wild-type SPOCK1 was constructed by PCR cloning. Briefly, full-length ORF encoding human SPOCK1 was PCR-amplified with Phusion High-Fidelity polymerase in two steps. For the first PCR, outer primers (F-out: 5′ GGCGGCGTGTGGCAGGAG 3′ and R-out: 3′ TAGAGAGCAACAATGGAGAAGAGACC 5′) were used in 30 cycles, using cDNA from HLE cells as a template. For the second PCR, inner primers (ORF-F: 5′ TTTTTGGATCCGAAATGCCTGCTATCGCGGTG 3′ and ORF-R: 3′ TTTTTCTCGAGCTACCATATGTACCCGACCTCATC 5′) were used, and the template was the purified product of the first PCR. Gel extracted fragment of the second PCR was inserted into a pcDNA™4/TO mammalian expression plasmid (Invitrogen, Carlsbad, CA, USA) using *Bam*HI and *Xho*I sites. The transfection was performed using Neon Transfection System (MPK5000, Life Technologies) with 2 × 10^5^ cells in a 100-µl pipette tip with 5 µg of SPOCK1 pcDNA™4/TO and empty vector pcDNA™4/TO using the following settings of the machine: 1,200 V, 50 ms, and 1 pulse. The cells were seeded in 6-well plates in DMEM supplemented with 20% FBS and 2 mM of l-glutamine. Stable transfectants were selected in a selective medium (complete growth medium supplemented with 500 µg/ml of Zeocin). Stably transfected monoclonal cell lines were generated by isolating individual colonies. The colony with the highest SPOCK1 expression was selected and expanded for further experiments.

### 2.6 Western Blotting

Cell cultures at 70%–80% confluency were harvested in lysis buffer containing 20 mM of Tris, 150 mM of NaCl, 2 mM of EDTA (pH 8), and 0.5% Triton X-100 supplemented with protease and phosphatase inhibitors (P8340). The protein concentration was measured using Protein Assay Dye Reagent Concentrate (500-0006, Bio-Rad, CA, USA). An equal amount of protein (20 µg) was separated by 10% sodium dodecyl sulfate (SDS)–polyacrylamide gel electrophoresis (TGX FastCast Acrylamide Kit, 10%, #1610173, Bio-Rad, CA, USA) and transferred to polyvinylidene difluoride (PVDF) membrane (IPVH00010, Merck Millipore, Burlington, MA, USA) using Trans-Blot Turbo Transfer System (1704150, Bio-Rad). The membrane was incubated with Blotting-Grade Blocker (#170-6404, Bio-Rad) for 2 h at room temperature and with primary antibody overnight at 4°C. After being washed three times in Tris-buffered saline containing 0.1% v/v Tween-20 (TBST containing 8.7 g of NaCl; 2.4 g of Tris; 0.05% Tween-20), the membrane was incubated with horseradish peroxidase (HRP)-conjugated secondary antibody for 1 h at room temperature, then washed three times in TBST, and visualized with SuperSignal™ West Pico PLUS Chemiluminescent Substrate (34580, Life Technologies). Images were performed using iBright FL1000 Imaging Systems (Life Technologies) and analyzed with ImageJ software.

### 2.7 Human Phospho-Kinase Array

Human Phospho-Kinase Array (ARY003B, R&D Systems, Minneapolis, MN, USA) was used for the detection of relative phosphorylation levels of 43 potential kinase phosphorylation sites. The assay was performed according to the manufacturer’s instructions, and 400 µg of protein of SPOCK1-silenced and control samples was used on each array. The signal detection was performed using iBright FL1000 Imaging Systems and analyzed with Carpentier G. Protein Array Analyze for ImageJ (2010) available online: http://rsb.info.nih.gov/ij/macros/toolsets/Protein Array Analyzer.txt

### 2.8 Bromodeoxyuridine Assay

Forty-eight hours after transfection, the culture medium was changed to fresh medium supplemented with 10 µM of bromodeoxyuridine (BrdU) (B5002, Merck KGaA) and incubated for 30 min at 37°C. The cells were fixed with ice-cold methanol for 10 min, washed three times with PBS, and incubated with 2 M of HCl for 10 min at room temperature. After being washed, the cells were incubated with an Anti-BrdU antibody for 1 h at room temperature. Finally, the cells were incubated with fluorescent-labeled Alexa Fluor 488 and DAPI (1:200) and mounted using Fluoromount. The stained slides were scanned with 3DHistech Pannoramic Confocal scanner and analyzed using CaseViewer CellQuant 2.2 (3DHISTECH Ltd., Budapest, Hungary).

### 2.9 Migration Assay

The cells were plated on coverslips and transfected with either SPOCK1 or scrambled siRNA as described before. Twenty-four hours after transfection, the cells were incubated with a complete growth medium containing 10 µg/ml of Mitomycin C (M5353, Merck KGaA, Darmstadt, Germany) for 3 h and then washed two times with PBS. The scratch was created using a 200-µl pipette tip. The debris was removed, and the cells were grown in a complete growth medium containing 1% FBS. The cells were fixed at 0-, 24-, and 48-h time points using methanol as described before and stained with H&E. The slides were scanned with Pannoramic P1000 scanner (3DHISTECH Ltd., Budapest, Hungary).

### 2.10 Invasion Assay

The invasiveness of the cell lines was studied using CytoSelect™ 24-Well Cell Invasion Assay (CBA-110, Cell Biolabs Inc., San Diego, CA, USA). The cells were transfected as described before; 24 h after transfection, 1 × 10^6^ cells were plated onto each insert. The assay was performed according to the manufacturer’s instructions.

### 2.11 Generation of Human Syndecan-1-Transgenic (hSDC^+/+^) Mice and Diethylnitrosamine-Induced Hepatocarcinogenesis

Diethylnitrosamine (DEN) hepatocarcinogenesis model was carried out as was described previously (9). Animal experiments were approved by the Ethics Committee of the Animal Health Care and Control Institute, Csongrád County, Hungary (protocol code XVI/03047-2/2008).

### 2.12 Immunohistochemistry

The surgical materials were collected and used according to the instructions of Semmelweis University Regional and Institutional Committee of Science and Research Ethics (TUKEB permit number: 155/2012), Medical Research Council Committee of Science and Research Ethics (permit number: 61303-2/2018/EKU). Human, mouse, rat liver, and human bone marrow formalin-fixed and paraffin-embedded tissue sections were deparaffinized and rehydrated. Heat-induced antigen retrieval was carried out by BOND Epitope Retrieval Solution 1 (AR9961, Leica Biosystems, Newcastle, UK). Endogenous peroxidase was blocked with 10% H_2_O_2_ in methanol for 20 min at room temperature. Sections were washed in TBST followed by blocking the non-specific protein binding with Novocastra Protein Block (RE71102, Leica Biosystems) for 10 min at room temperature. After a wash step, the sections were incubated with SPOCK1 and CHD1L primary antibodies overnight at 4°C. The next day, the sections were washed and incubated with HISTO-Labeling System for 30 min at room temperature (30011.R500, Department of Immunology and Biotechnology, Pécs, Hungary). The reactions were visualized using diaminobenzidine 1:50 (ImmPACT DAB, SK-4105, Vector Laboratories, Burlingame, CA, USA) followed by counterstain with hematoxylin. The sections were dehydrated and covered using BIOMOUNT (BMT-500, BIOGNOST D.O.O, Zagreb, Croatia). Stained slides were scanned at ×20 magnification with Pannoramic P1000 scanner, and the quantitative analysis was performed using CaseViewer DensitoQuant 2.2 (3DHISTECH Ltd., Budapest, Hungary).

### 2.13 Automated Western Blotting System (WES™)

The samples were analyzed using WES™ (ProteinSimple, San Jose, CA, USA, a Bio-Techne Brand) automated capillary-based electrophoresis instrument. Cell lysates were prepared as described at the Western blotting and were further diluted in 0.1× Sample Buffer (ProteinSimple, 042-195), and the concentration was set to 1 µg/µl. Conditioned media were collected and concentrated using 10,000 NMWL centrifugal filters (Amicon Ultra-4, UFC801024, Merck Millipore, Burlington, MA, USA). Fluorescent Master Mix was added to the samples in a 1:4 ratio followed by 5-min incubation at 95°C. The samples, blocking reagent (Antibody Diluent; 042-203, ProteinSimple), diluted primary antibody (see **Supplementary Table 1**), secondary antibody (042-206, ProteinSimple), chemiluminescent substrate, and wash buffer were loaded into WES capillary plate according to the manufacturer’s instruction. Five micrograms of protein from cell lysate and 5 µl of conditioned medium were separated using 12–230 kDa of Separation Module (SM-W004, ProteinSimple) with the following settings: separation (395V, 30 min), blocking (5 min), incubation with primary antibody (30 min), incubation with secondary antibody (30 min), and chemiluminescent detection (15 min). The results were analyzed, and the pictures were edited using Compass software (San Jose, CA, USA).

### 2.14 Quantitative Real-Time PCR

Total RNA was isolated using RNeasy Plus Mini Kit (74134, Qiagen, Germany). For reverse transcription, 1 µg of RNA was used, and the assay was performed with High Capacity cDNA Reverse Transcription Kit (4368814, Life Technologies) according to the manufacturer’s instructions. The mRNA level was quantified using a SPOCK1 TaqMan Gene Expression Assay (assay ID: *Hs00270274_m1*; Life Technologies) and TaqMan Fast Advanced Master Mix (4444556, Life Technologies) with the following protocol: 2 min, 50°C UNG incubation; 2 min, 95°C polymerase activation; 40 cycles of 1 s, 95°C denaturation; and 20 sec, 60°C annealing and elongation. The relative quantification was normalized to endogenous β-actin (4326315E, Life Technologies) expression.

### 2.15 Hepatocyte Isolation

Hepatocytes were isolated from the liver of F344 rats. The breeding animals were purchased from Charles River Laboratories (Écully, France). The animals were housed in plastic cages (556 × 334 mm, AnimaLab, Poznań, Poland) and kept under standard conditions: 12 h of light–dark cycles, constant temperature (23°C), and humidity (22%). Standard rodent chow (V1535000, SSNIFF, Soest, Germany; 15-mm pellets) was provided *ad libitum*.

Hepatocyte isolation was performed using a two-step collagenase perfusion method as described before (31). The cells were filtered through a 100-µm nylon filter, and then the suspension was centrifuged 3 times for 5 min at 50*g* to separate hepatocytes from non-parenchymal cells. The cells were kept on ice for 6 h. Hepatocytes were plated on glass slides every hour for immunocytochemistry. The smears were air-dried and then fixed with ice-cold methanol for 10 min. SPOCK1 fluorescent immunocytochemistry was performed as described before.

### 2.16 Statistical Analysis

Statistical analysis was calculated, and bar charts were drawn using the GraphPad Prism v8.01 (GraphPad Software, La Jolla, CA, USA) software. Results were analyzed by unpaired Student’s *t*-test, and significance was defined as *p < 0.05, **p < 0.01, and ***p < 0.001.

## 3 Results

### 3.1 SPOCK1 in Healthy, Cirrhotic, and Tumorous Human Livers

**Figure 1:**
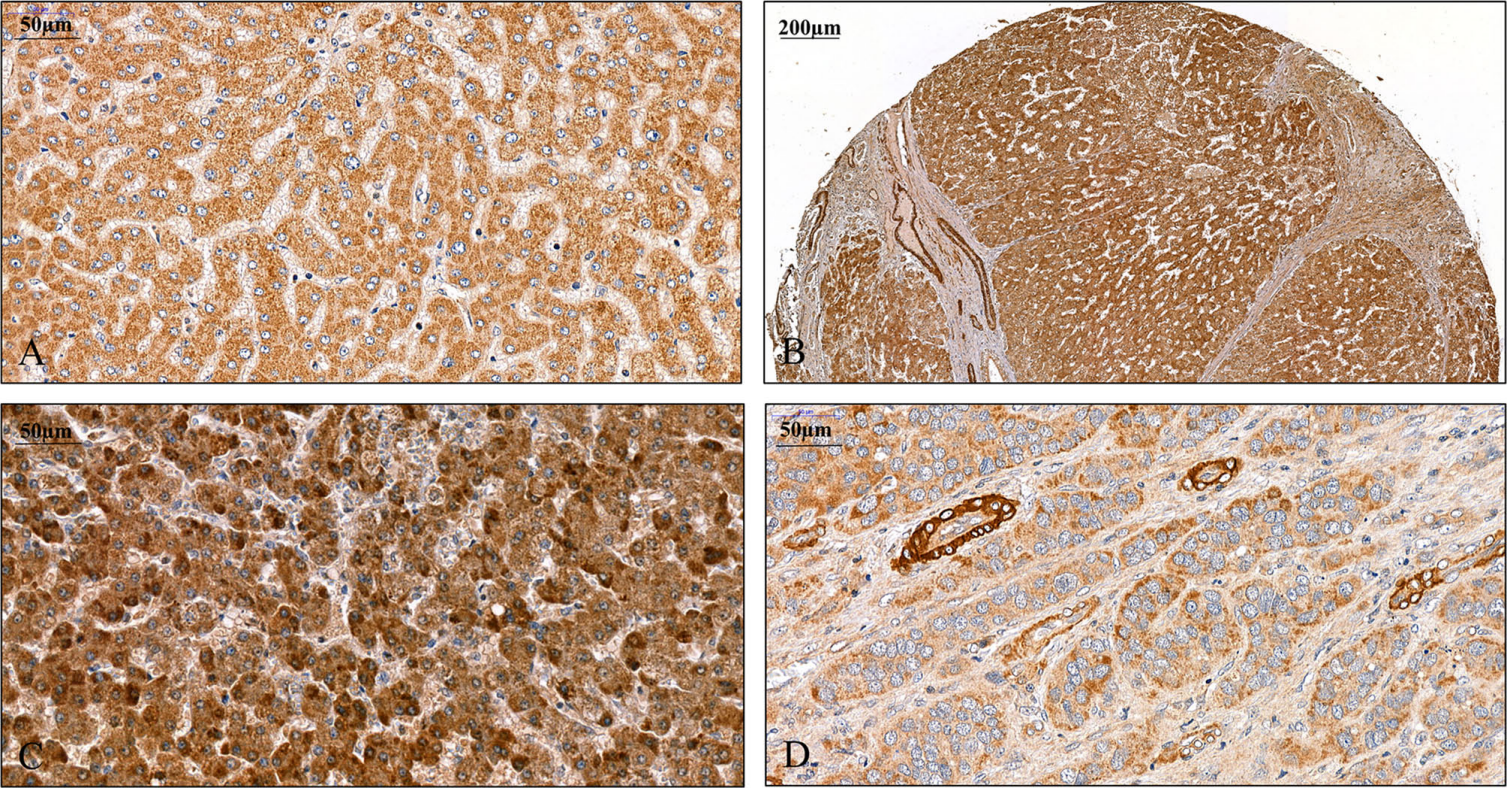
Detection of SPOCK1 protein on human liver specimens. **(A)** Normal liver, moderate staining in the cytoplasm of hepatocytes. **(B)** Cirrhotic liver with intensive staining in the cytoplasm. Endothelial cells of the blood vessels also show the presence of high levels of SPOCK1. Areas of connective tissue are devoid of SPOCK1. **(C)** Hepatocellular carcinoma, the tumor cells are loaded with the proteoglycan. **(D)** Hepatocellular carcinoma, the staining intensity is modest in tumor cells, whereas the cytoplasm of endothelial cells shows high protein levels.

**Figure 2:**
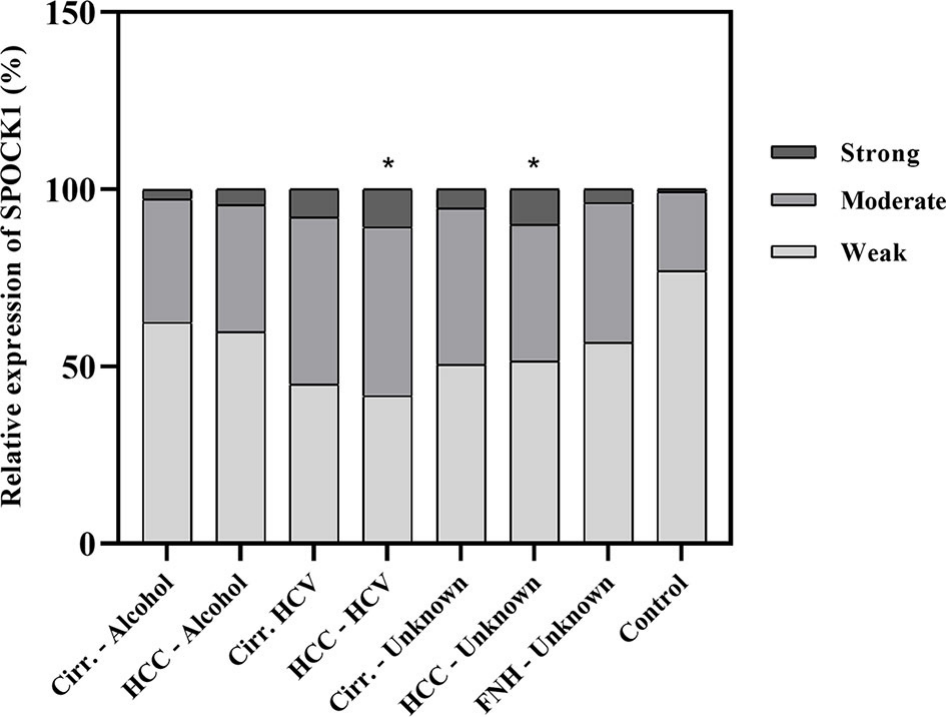
Quantitative assessment of the expression of SPOCK1 protein in liver cirrhosis and cancer of various origins. Significant increase in cancer specimens. n = 76, *p < 0.05.

A pilot survey of a few human liver specimens revealed modest cytoplasmic positivity in the hepatocytes of seemingly healthy human livers. While SPOCK1 expression was high in the cirrhotic hepatocytes, the staining intensity in cancer cells was heterogeneous. The cytoplasm of the endothelial cell displayed intense cytoplasmic positivity; however, except for the blood vessels, the connective tissue was negative. SPOCK1 reaction in the liver cells was an unexpected finding. Apparently, although the expression of SPOCK1 is very low in healthy hepatocytes, their injuries facilitate the upregulation of the proteoglycan, and its expression increases in liver cirrhosis and hepatocellular carcinoma. SPOCK1 expression was assessed on human liver cirrhosis and hepatocellular carcinoma of various etiologies. Compared with control livers, significant upregulation could be detected in both hepatitis C virus (HCV)-infected cirrhotic and tumorous livers (**Figures 1**, **2**).

**Figure 3:**
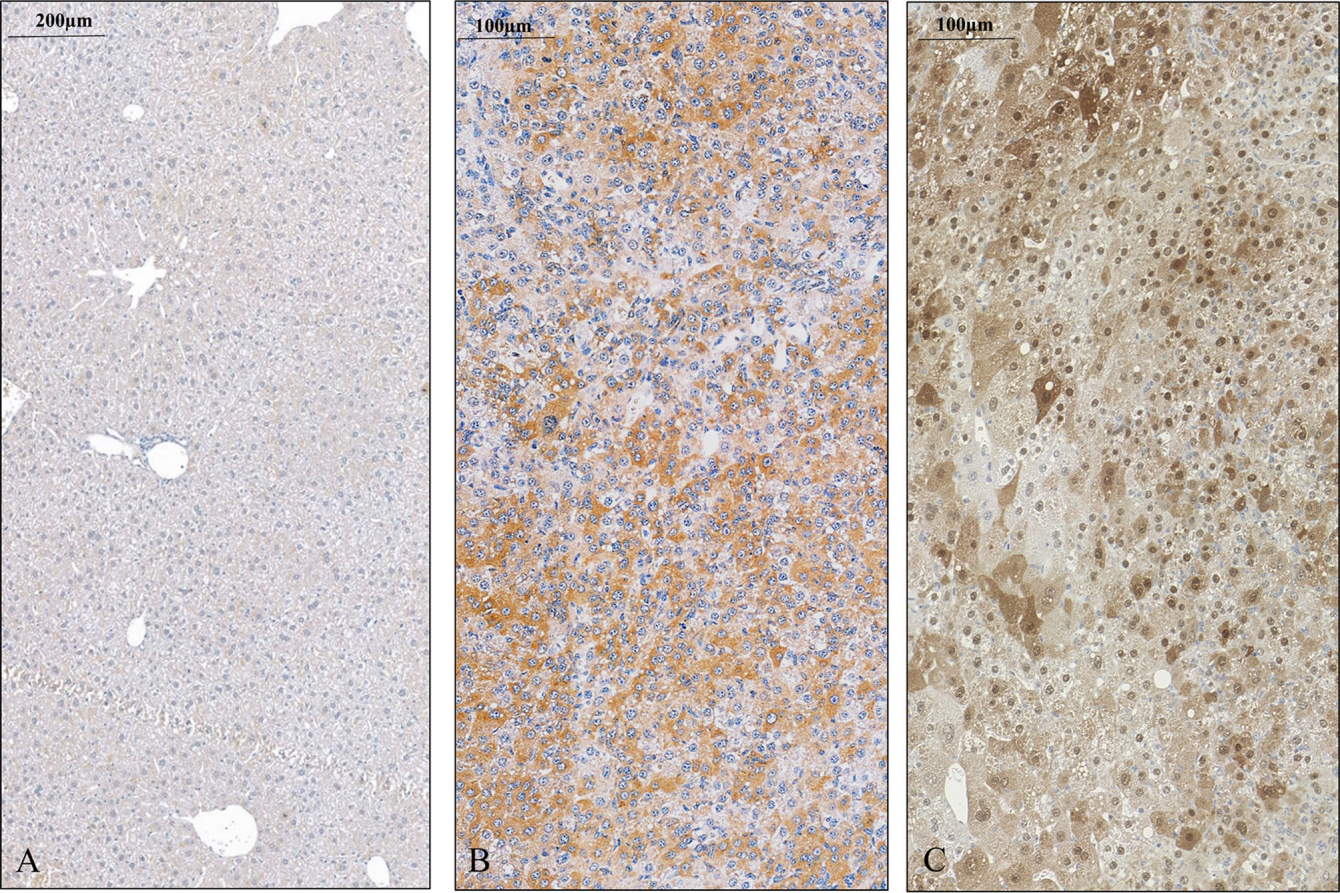
SPOCK1 expression in control mouse liver and hepatocellular cancer. **(A)** Healthy liver with low amounts of SPOCK1 around the central veins. **(B)** SPOCK1 detection in diethylnitrosamine (DEN) induced hepatocellular cancer and transformed liver cells that contain ample amounts of proteoglycan. **(C)** Expression of CHD1L, the transcription factor of SPOCK1, in the same liver. The protein is detectable in both the nucleus and the cytoplasm.

In the next step, an experimental hepatocarcinogenesis model was designed, by utilizing the genotoxic diethylnitrosamine as a proxy carcinogen to follow the changes of SPOCK1 during the process of tumor development (**Figure 3**). The results provided clear evidence that SPOCK1 is hardly detectable in the liver of 6-month-old healthy mice. Weak cytoplasmic staining of the hepatocytes could be deciphered only around the blood vessels. On the contrary, SPOCK1 was well detectable in the transformed hepatocytes without fatty change. These findings support the hypothesis that even if SPOCK1 expression is very low in the normal liver, it is upregulated during carcinogenesis. Furthermore, the process was facilitated by the upregulation of CHD1L, a known oncogenic factor of the proteoglycan (32).

### 3.2 SPOCK1 Expression Is Rapidly Activated in Isolated Hepatocytes

SPOCK1 expression was also studied in healthy rat livers. Similar to that in the mouse liver, the proteoglycan was hardly detectable. To evaluate if cellular stress activates the synthesis of the proteoglycan in normal hepatocytes or it is the sign of impaired secretion, hepatocytes were isolated from rat livers, and the expression of SPOCK1 was followed in time.

**Figure 4:**
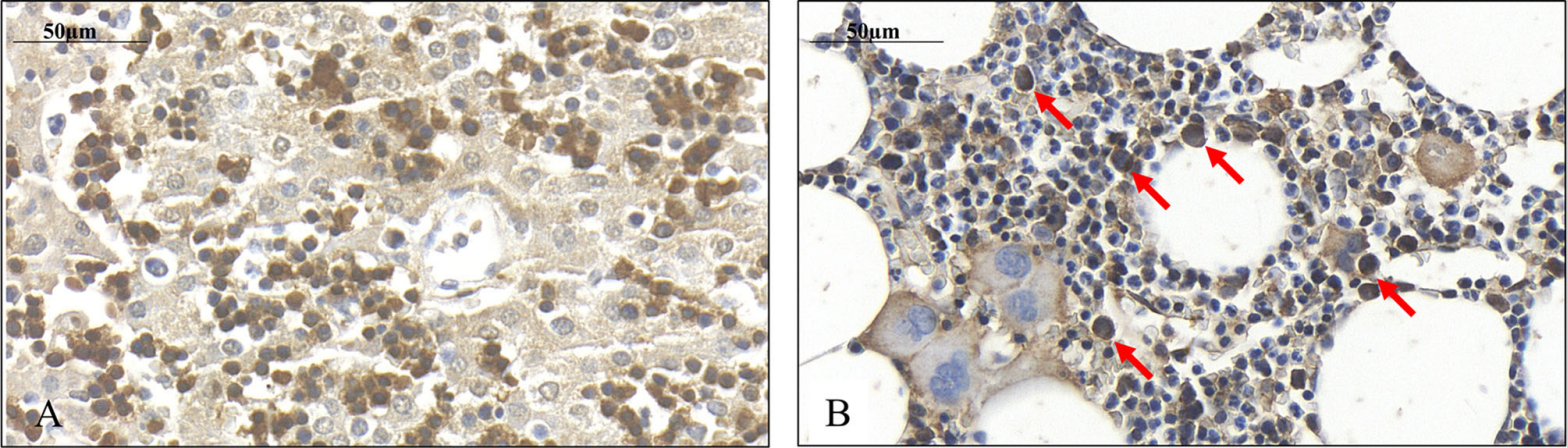
Presence of SPOCK1 in fetal human liver. **(A)** The weak cytoplasmic staining exhibits a good correlation with SPOCK1 intensity in normal mouse liver; normoblasts in the sinusoids display strong staining. **(B)** SPOCK1-positive normoblasts are detectable in the bone marrow marked with arrows.

**Figure 5:**
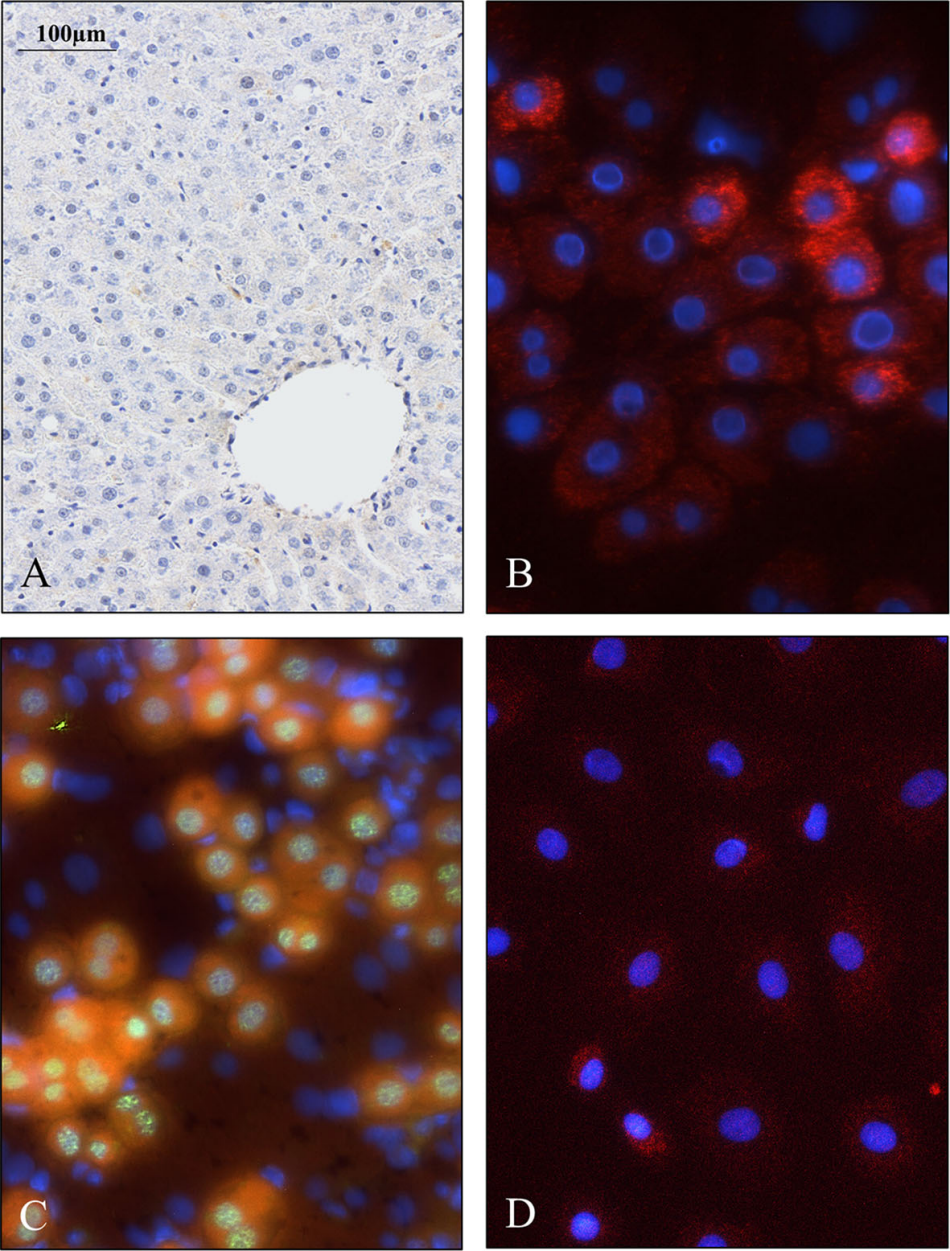
Weak SPOCK1 staining in healthy rat liver, fast upregulation of SPOCK1 during hepatocyte isolation. **(A)** SPOCK1 protein cannot be detected in healthy rat liver. **(B, C)** Upregulation of SPOCK1 as a function of time in isolated hepatocytes. **(C)** HNF4α (Hepatocyte Nuclear Factor 4 *Alpha*) can be detected in the nucleus. **(D)** After 3 days of culture, the proteoglycan expression returns to the original low levels.

Immediately after isolation, a proportion of the cells displayed wide cytoplasm with weak SPOCK1 positivity. Nevertheless, in some cells with similar nuclei, the cytoplasm shrank, exhibiting intensive SPOCK1 positivity. Over time, the number of these SPOCK1-positive cells kept increasing, whereas their cytoplasm was shrinking. After plating, the intensity of SPOCK1 expression gradually decreased, and by the end of the third day, it turned back to the state observed immediately after isolation (**Figure 4**). Taken together, SPOCK1 expression in human fetal livers was comparable with that of normal mouse and rat livers, whereas normoblasts in the sinusoids and the bone marrow were strongly positive (**Figure 5**).

### 3.3 SPOCK1 Localizes in the Mitochondria of Human Hepatoma Cell Lines

**Figure 6:**
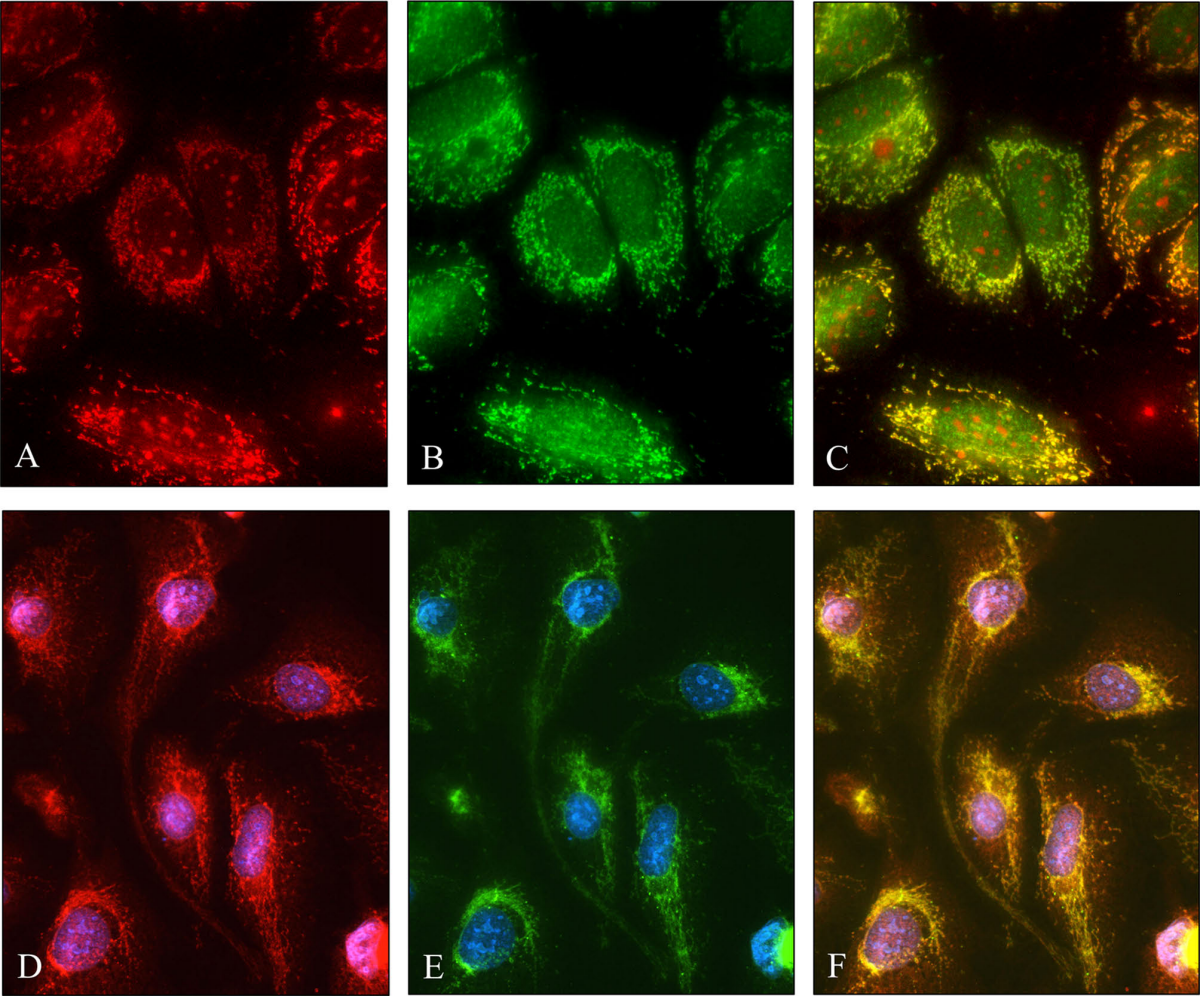
Colocalization of SPOCK1 with a mitochondrion marker and TOMM20 mitochondrial protein. **(A)** Staining of HuH7 hepatoma cells with MitoTracker. **(B)** SPOCK1 alone. **(C)** The MitoTracker-red and SPOCK1 (green) stainings are aligned. **(D, E)** Immunostaining of HLE cells with SPOCK1 (red) and TOMM20 (green); the two proteins are fully aligned **(F)**. Original magnification, ×40.

The granular cytoplasmic SPOCK1 reaction raised the possibility that the proteoglycan localizes in the mitochondria; thus, double fluorescent immunostainings were carried out with SPOCK1 antibody together either with TOMM20 or mitochondrial marker (MitoTracker-red, M22425) on hepatoma cell lines (**Figure 6**). We observed that SPOCK1 was present in the mitochondria, and it colocalized with both MitoTracker and TOMM20 (Translocase of Outer Membrane of Mitochondrion). This finding indicates that SPOCK1 belongs to the proteins that TOMM20 is ready to transfer from the cytoplasm to the mitochondria.

### 3.4 Functional Assays to Detect the Effect of SPOCK1 Silencing

**Figure 7:**
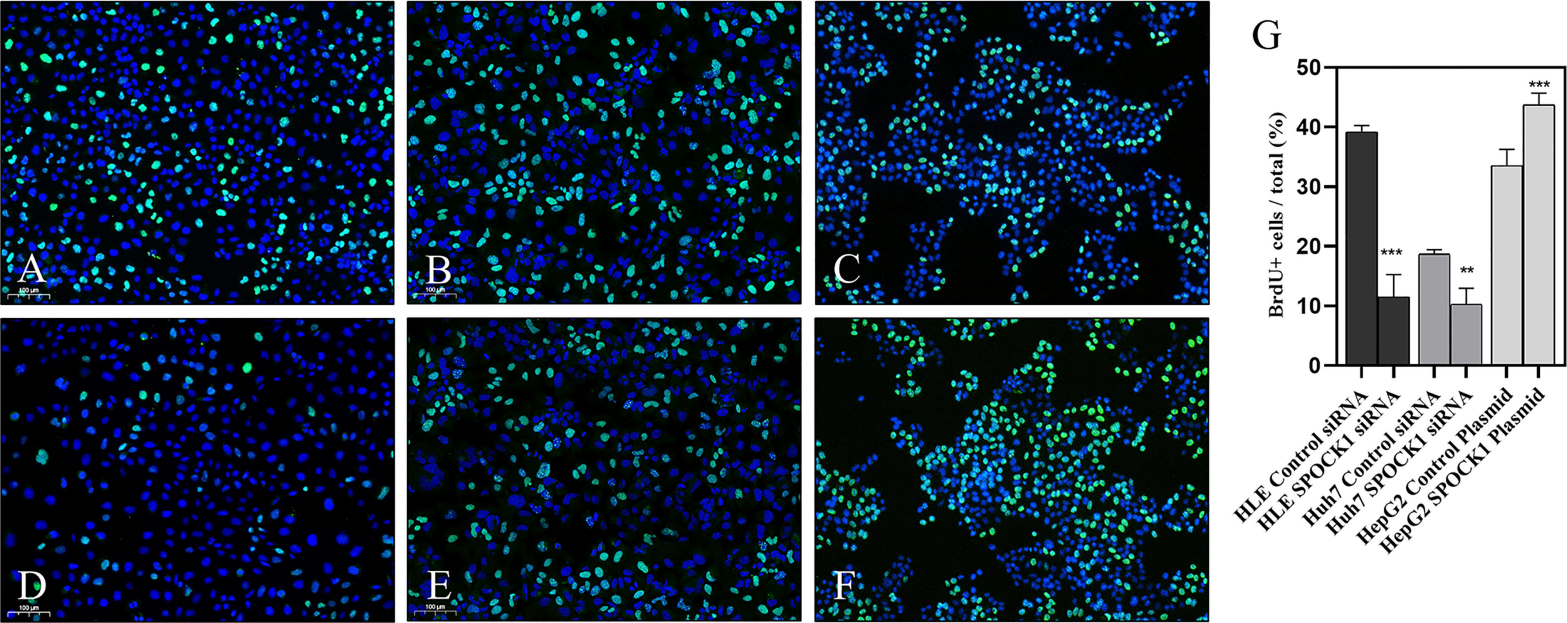
BrdU uptake was studied in HLE and HuH7 cells after silencing SPOCK1 and HepG2 cells overexpressing the proteoglycan. Silencing of SPOCK1 inhibits its upregulation by transfection, promoting BrdU uptake. **(A)** HLE, **(B)** HuH7, and **(C)** HepG2 control cell lines. **(D)** HLE and **(E)** HuH7 cell lines transfected with siRNA. **(F)** HepG2 cell line transfected with the SPOCK1 expression vector. **(G)** Quantitation of the fluorescent nuclei. Green BrdU, blue DAPI. Original magnification, ×20. **p < 0.01, ***p < 0.001.

To assess the role of SPOCK1 in the regulation of cell proliferation, its effect on DNA synthesis was investigated by BrdU uptake. The amount of the incorporated thymidine analog indicated the activity of DNA synthesis in tumor cells. Control cells were compared with SPOCK1 siRNA silenced and SPOCK1 expression vector-transfected tumor cells following BrdU uptake for 30 min. As an effect of SPOCK1 inhibition, the number of labeled cell nuclei decreased to 25% and 50%, respectively, in siRNA-treated HLE and HUH7 cells and increased to about 130% in SPOCK1-transfected HepG2 cells (**Figure 7**).

**Figure 8:**
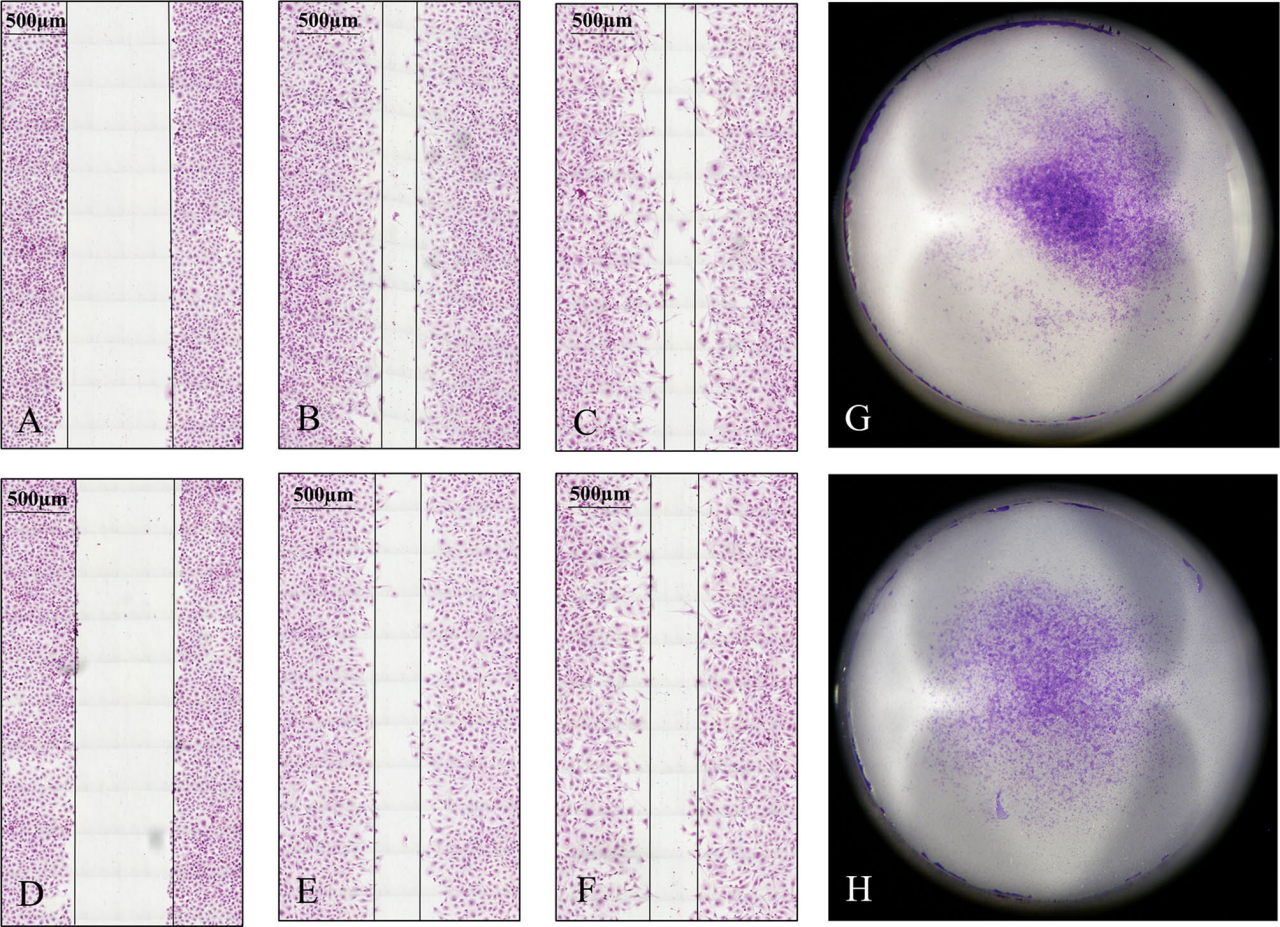
Migration and invasion assays of HLE cells. The tumor cells were observed for 48 h in the migration assay. Silencing of SPOCK1 by siRNA inhibited the migration of the tumor cells. **(A–C)** Control cells at 0, 24, and 48 h time points. **(D–F)** SPOCK1 siRNA transfected cells at 0, 24, and 48 h time points. Invasion assay of control **(G)** and SPOCK1-silenced **(H)** HLE cells. Silencing inhibited both the migration and invasion of the tumor cells. As control Huh7 cells neither migrate nor invade collagen, no difference between the control and silenced cells could be detected.

Migration and invasion of HLE and Huh7 tumor cells in control and SPOCK1 siRNA silenced samples showed that siRNA inhibited both migration and invasion of HLE cells. On the contrary, the differentiated control Huh7 cells neither migrate nor invade collagen as it was already determined previously. Thus, we could not detect any difference with these methods between its control and silenced cells (**Figure 8**).

### 3.5 Effect of SPOCK1 Silencing on Regulatory Proteins of Hepatoma Cell Lines

#### 3.5.1 Phosphokinase Arrays

**Figure 9:**
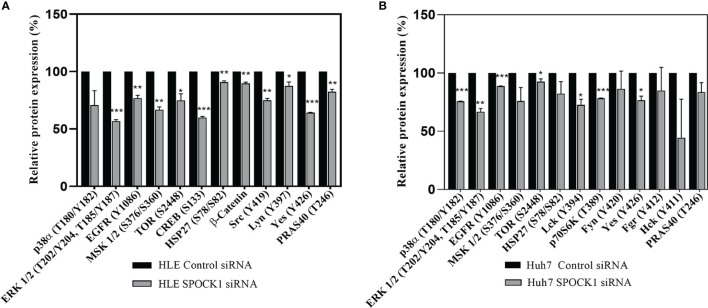
Phosphotyrosine kinase arrays of HLE **(A)** and Huh7 **(B)** cells showing the changes as an effect of SPOCK1 silencing by siRNA. Downregulation of SPOCK1 was accompanied by decreased activation of EGFR, and MAPK pathways in both HLE and HuH7 cell lines together with several members of the Sarc family. The inhibition was more effective in the highly aggressive HLE cell line where MSK1, Creb, mTOR, and β-catenin were also affected. *p < 0.05, **p < 0.01, ***p < 0.001.

HLE and HuH7 hepatoma cells were transfected with SPOCK1 siRNA. Subsequently, their cell homogenates were incubated with the premade filters containing specific antibodies against phosphokinase proteins. SPOCK1 silencing significantly downregulated the activated EGF receptor in both HLE and HuH7 cell lines, together with decreased phosphorylation of ERK1/2, MSK1/2, and CREB (S133), observed in the MAPK pathway. Additionally, 3 proteins from the SRC family, Src, Lyn, and Yes were also downregulated in HLE cells. The EGFR downregulation resulted in significant inhibition of both the ERK and p38 pathways and different Src family members in the Huh7 cells. Inhibition of the mTOR pathway was less detectable in the two cell lines (**Figure 9**).

#### 3.5.2 Western Blotting

**Figure 10:**
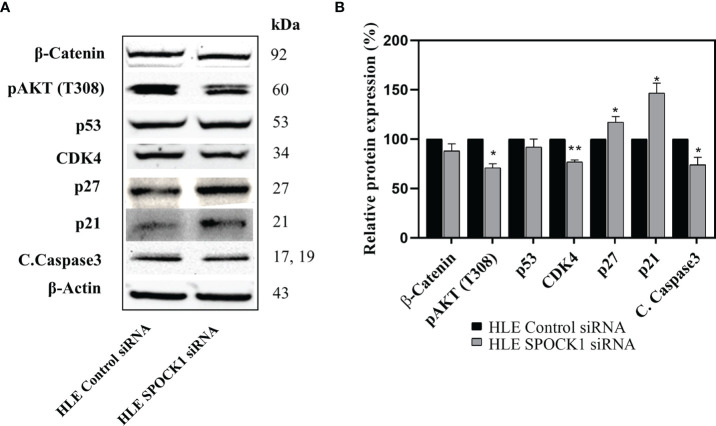
Western blotting of control and SPOCK1 siRNA silenced HLE cells. **(A)** Additional consequences of SPOCK1 downregulation. Inhibition of the proteoglycan resulted in the downregulation of pAkt (T308), and CDK4 cyclin-dependent kinase, accompanied by the elevated expression of p21 and p27 cyclin-dependent kinase inhibitors. **(B)** Quantitative evaluation. *p < 0.05, **p < 0.01.

Besides those, as revealed on the phosphokinase array, SPOCK1 silencing influenced several other regulatory proteins. Phospho-Akt(T308), CDK4, and cleaved caspase-3 expression decreased, while the expression of cyclin-dependent kinase inhibitors p21 and p27 increased. The modest inhibition of cleaved caspase-3 indicated that SPOCK1 can interfere with the apoptotic machinery (**Figure 10**).

#### 3.5.3 Detection of SPOCK1 After Silencing or Transfection of Hepatoma Cell Lines

**Figure 11:**
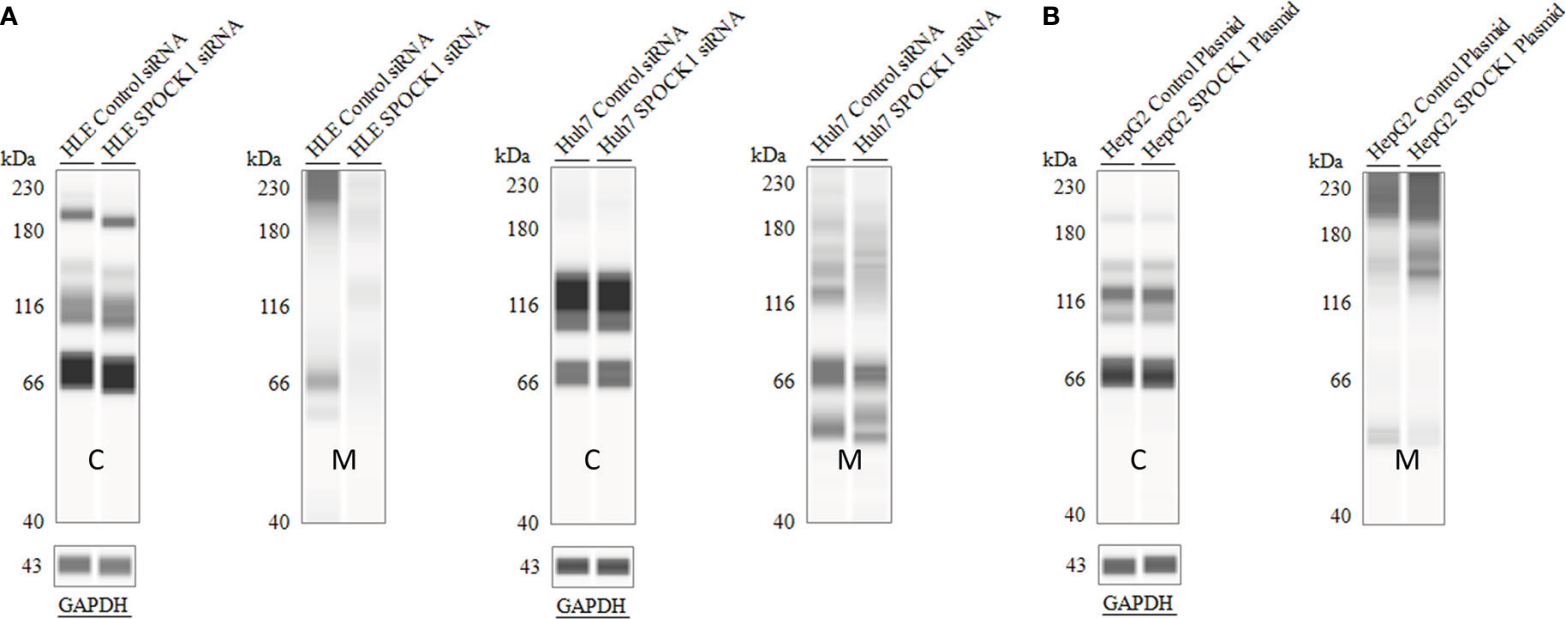
SPOCK1 downregulation and upregulation were mirrored only by the amount of the secreted proteoglycan. WES of SPOCK1-silenced **(A)** and SPOCK1-transfected **(B)** cells were separated and run parallel with the part of the proteins secreted into the culture medium. The intracellular concentration of the protein remained constant, and only the amounts secreted to the medium represented the changes. C, tumor cells; M, culture medium.

SPOCK1 mRNA decreased in SPOCK1-silenced and increased in SPOCK1-transfected hepatoma cell lines. However, intracellular SPOCK1 protein expression was constant, and it was only the culture medium where the effect of downregulation or upregulation could be detected on the protein level (**Figure 11** and **Table 1**).

**Table 1:** Downregulation of SPOCK1 mRNA in HLE and Huh7 cells by SPOCK1 siRNA and its upregulation after transfection by SPOCK1 construct of HepG2.

Cell line	Relative quantification (fold change)	95% CI
HLE	0.07	0.046–0.109
Huh7	0.292	0.139–0.613
HepG2	30.517	16.698–55.771

Data indicate the fold changes.

### 3.6 SPOCK1 and Syndecan-1 Compete in the Liver and in Hepatocellular Cancer

**Figure 12:**
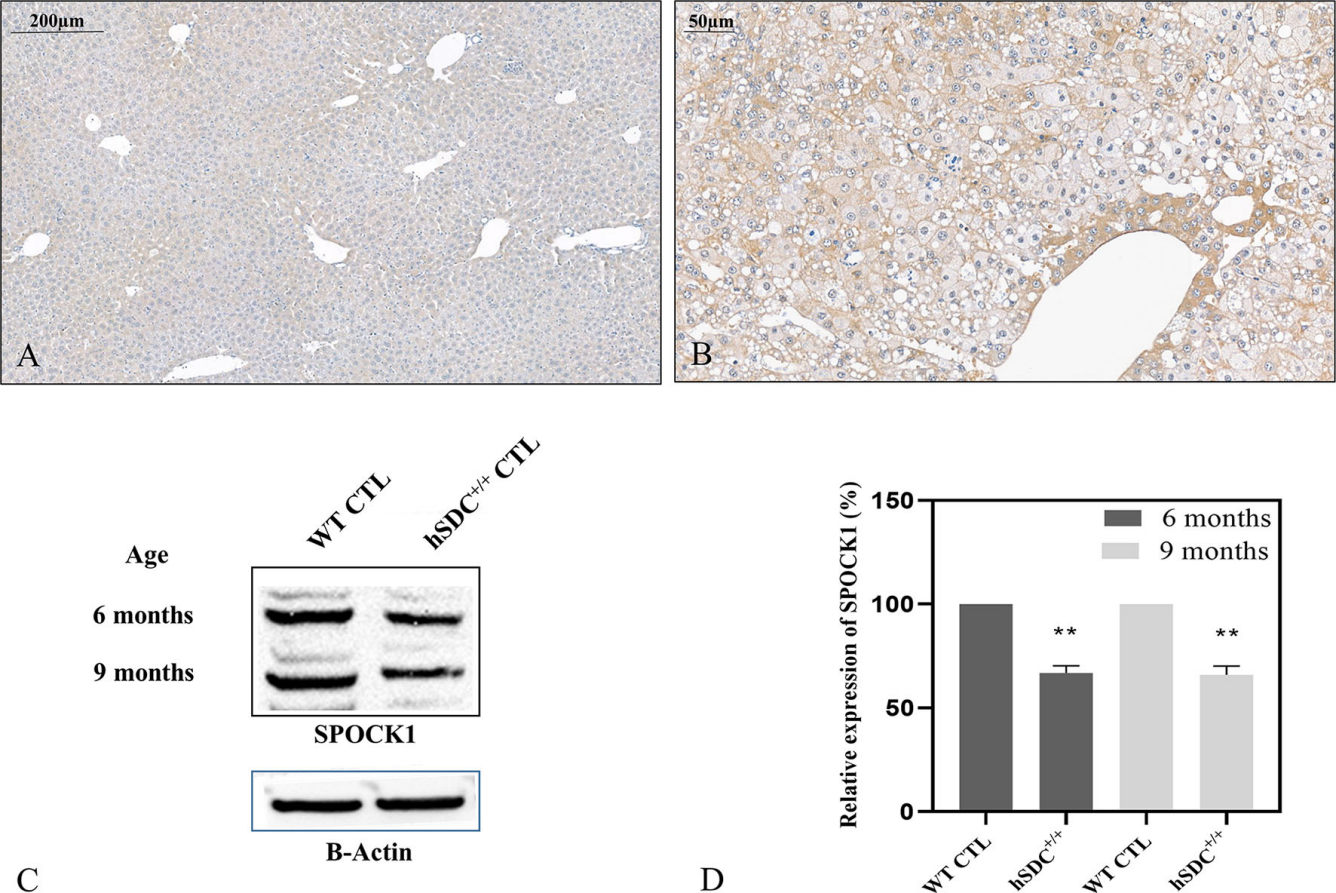
SPOCK1 expression in hSDC-1^+/+^ transgenic control liver and its diethylnitrosamine (DEN)-induced cancer. **(A)** Weak SPOCK1 positivity was detected around the central veins in hSDC-1^+/+^ control liver. **(B)** Hepatocellular cancer 11 months after DEN exposure in the hSDC-1^+/+^ liver. The intensity of SPOCK1 staining is still modest, except around the central veins. Western blotting **(C, D)** supports the results of immunohistochemistry; SPOCK1 expression in hSDC-1^+/+^ control livers is lower than that in wild-type liver throughout the whole experiments. **p < 0.01.

**Figure 13:**
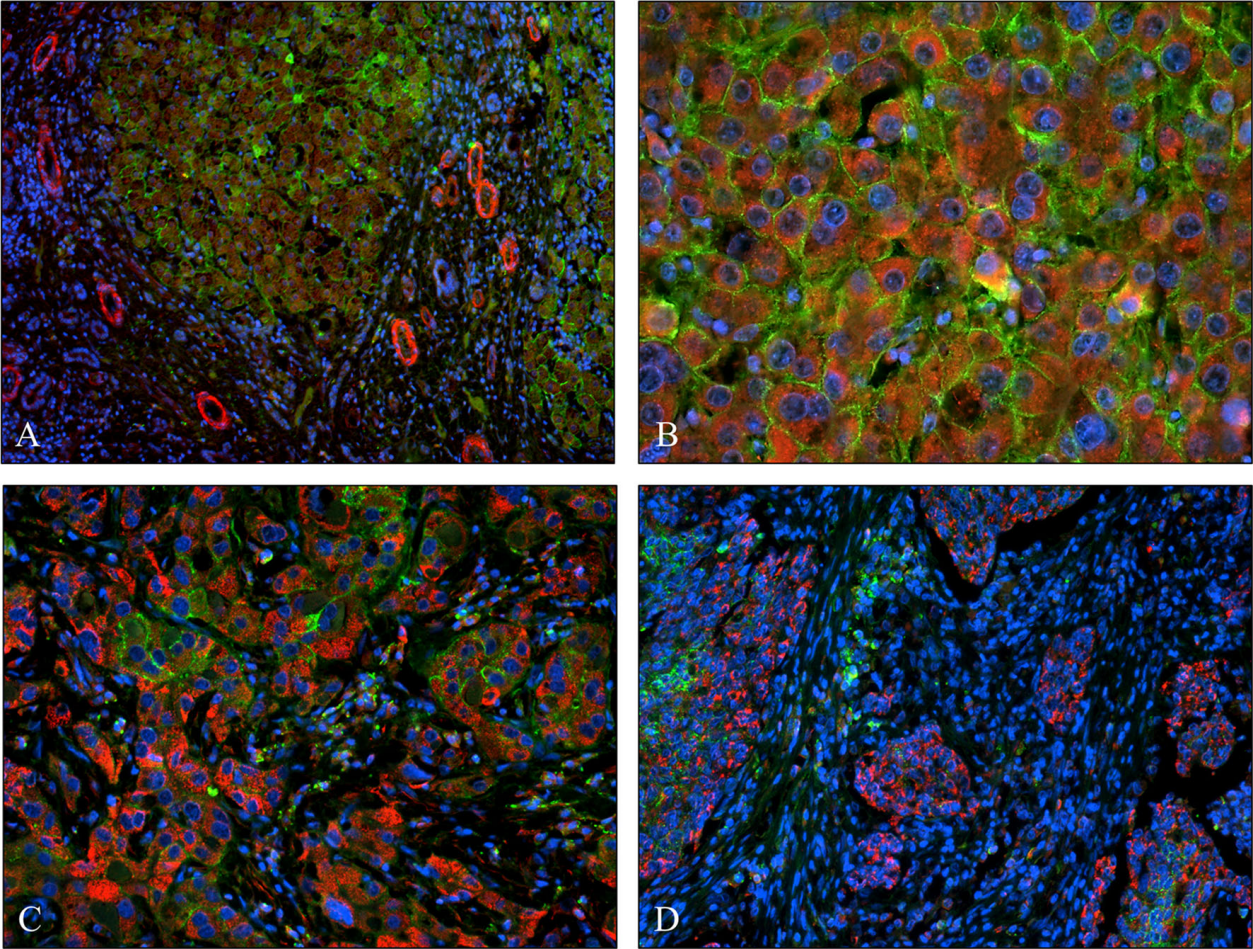
Co-expression of syndecan-1 and SPOCK1 in human liver cirrhosis and hepatocellular cancers. **(A)** Cirrhotic remodeling of the liver structure. A modest amount of SPOCK1 in the cytoplasm of hepatocytes (red), with syndecan-1 on the surface of the same cells (green). SPOCK1 containing endothelial cells of blood vessels are present in the stroma. **(B)** Well-differentiated hepatocellular cancer (HCC) with intensive cytoplasmic SPOCK1 staining and large amounts of syndecan-1 on the surface of tumor cells. **(C)** HCC with de-differentiated cancer cells. Intensive SPOCK1 expression in the cytoplasm; syndecan-1 vanished from the majority of cancer cells. **(D)** Micro metastases of HCC migrating in the tumor stroma. While SPOCK1 can be detected in large amounts, syndecan-1 protein cannot be observed.

We studied if the presence or overexpression of syndecan-1 the major transmembrane heparan sulfate proteoglycan of the liver can interfere with the tumor-induced upregulation of SPOCK1. Without DEN exposure, no difference could be detected between wild type and hSDC^+/+^ by immunostaining (**Figures 3A**, **12A**). However, Western blotting revealed that hSDC^+/+^ transgenic control livers expressed a lower amount of SPOCK1 (**Figures 12C, D**). Similarly, hSDC^+/+^ tumors contained less SPOCK1 than tumors developed in wild-type livers (**Figures 3B**, **12B**). In normal human liver, syndecan-1 and SPOCK1 are well separated, with the former being detected on the cell surface of hepatocytes and the latter in their cytoplasm. In contrast, in human liver cancer, syndecan-1 expression decreases, or even disappears, whereas the cytoplasm is filled with SPOCK1 (**Figure 13**).

## 4 Discussion

### 4.1 Uncertainties Related to SPOCK1

Although SPOCK1 was discovered in 1997, the first reports related to its oncogenic potential were published after 2010, describing the implication of the proteoglycan in gastric cancer (33). In the past 10 years, many more accounts have called attention to the oncogenic potential of SPOCK1. However, it is still uncertain if SPOCK1 has physiological functions outside the central nervous system and where it localizes *in vivo*. Is it an ECM protein? Or similar to serglycin (34), can it be detected in the cells of epithelial origin? The first assessment of the role of SPOCK1 in tissue metabolism was published last year. According to this report, SPOCK1 regulates adipogenesis-related genes, participates in thermoregulation, induces adipocyte differentiation, and is likely to be involved in the development of obesity and type 2 diabetes (35). Furthermore, SPOCK1 transgenic mice develop fatty liver.

Our work indicates that except for endothelial cells of blood vessels, neither hepatocytes nor the connective tissue of the healthy liver expresses abundant amounts of SPOCK1. However, our results demonstrated that intensive cytoplasmic SPOCK1 expression occurs in normal hepatocytes after their isolation, which most probably is related to cellular stress. The support of these presumptions needs further evaluation. This is in contrast with a recent report emphasizing that SPOCK1 is expressed presumably in activated hepatic stellate cells (HSCs). However, in the published paper, SPOCK1 immunostaining was localized in the cytoplasm of hepatocytes (30, 36), whereas the connective tissue was almost free of immunoreaction. Our studies on human liver specimens support the notion that SPOCK1 is upregulated in cirrhotic liver and hepatocellular carcinoma, but the localization is not stromal. Cultured HSCs do express SPOCK1 in their cytoplasm (36), and we could confirm this in LX2 immortalized HSC cells.

### 4.2 SPOCK1 in Human Tumors, Intracellular or Stromal?

Immunohistochemical analysis of 76 surgically removed liver specimens revealed that the expression of SPOCK1 is a characteristic feature of human liver cirrhosis and cancer, and its accumulation occurs in the cytoplasm of hepatocytes and the cells of hepatocellular carcinoma. The most significant increase was observed in hepatitis C-infected cirrhotic and tumorous specimens. In general, several tumors of epithelial origin express SPOCK1 in the cytoplasm, including prostate cancer (36, 37), breast cancer (38), gastric cancer (22), and several others (27). In the case of non-small cell lung cancer, the presence of SPOCK1 contributes to the resistance of the third-generation tyrosine kinase inhibitor (39). SPOCK1 can also be the regulator of brain metastasis of lung cancers (40), raising the opportunity to use the protein as a predictive tumor marker. Taken together, SPOCK1 is claimed to be an oncogene, involved in major oncogenic events, such as cell cycle regulation, DNA synthesis, migration, invasion, and EMT (27). This summary supports our findings presented here.

### 4.3 SPOCK1 and Liver Cancer

Thus, our results on the human hepatocellular cancer (HCC) and mouse hepatocarcinogenesis models, together with literature data, provided confirmation for the presence of SPOCK1 in diseased hepatocytes of cirrhotic liver and cancer cells (29). In support of earlier studies, the transcription factor of SPOCK1 was also upregulated in the experimental hepatocarcinogenesis model. However, the expression of cytoplasmic SPOCK1 in seemingly normal human livers needs explanation, especially as this finding is the opposite of what we found in healthy rodent livers. Our hypothesis is that during the significantly increased lifespan, the human liver is exposed to stressful injuries, capable of upregulating the proteoglycan. Indeed, the expression of SPOCK1 in the cytoplasm of fetal human livers was comparable with that of normal mouse and rat livers. In stress situations in our experiments, isolated hepatocytes from rat livers exhibited rapid transient upregulation of SPOCK1 in the cytoplasm.

### 4.4 *In Vitro* Models

HLE, HuH7, and HepG2 cell lines express considerable amounts of SPOCK1 in the cytoplasm, mainly localized in the mitochondria. At this point, it is uncertain if it interferes with the mitochondrial function of these hepatoma cells. The potential of SPOCK1 to inhibit apoptosis has been published (29, 41), and this function of SPOCK1 is related to the effect of its transcription factor CHD1L (42).

To detect the mechanistic function of SPOCK1, we followed the migration and invasion in the HLE and Huh7 cell lines after silencing with SPOCK1 siRNA. Both migration and invasion were successfully inhibited in the highly aggressive HLE cell line, but Huh7 cells neither migrate nor invade collagen. The mechanism behind this phenomenon needs further evaluation. To investigate if SPOCK1 has the potential to interfere with DNA synthesis, we downregulated its expression with siRNA or, alternatively, overexpressed it by using a SPOCK1 construct. From these assays, we learned that the proteoglycan interfered with the DNA synthesis, as its downregulation hampered the incorporation, whereas its elevated amount supported the uptake of BrdU. SPOCK1 inhibition pointed to CREB and CDK4, as both proteins were downregulated by SPOCK1 silencing, suggesting that they regulate the proteoglycan (43). So far, no report has evaluated the relationship between CREB and SPOCK1. However, as more than 4,000 proteins contain creb binding cre element in their promoters, SPOCK1 is likely equipped with this feature (44, 45). The CREB transcription factor is regulated *via* several tyrosine kinase receptor-activated pathways, and SPOCK1 silencing resulted in decreased EGFR activation followed by downregulation of the MAPK pathway (45). This explains not only the decreased action of CREB but also the lower levels of CDK4 cyclin-dependent kinase. These, together with the elevation of p21 and p27 cyclin-dependent kinase inhibitors, support the notion that SPOCK1 downregulation inhibits proliferation (46). One so far unknown consequence of SPOCK1 silencing is the inhibition of the members of the Src family. This family contains several non-receptor tyrosine kinase proteins, known to be implicated in cancer progression (47) including their involvement in migration, invasion, and proliferation. The HBX protein of the hepatitis B virus needs the action of Src to activate the signaling of RAS (48). C-Src is frequently upregulated in liver cancer and promotes the upregulation of the Hippo pathway (49). As SPOCK1 silencing inhibits the activation of Src, it is possible that this function similarly affects the Hippo pathway. In addition, it was recently reported that the Src protein in the liver interferes with the mitochondrial OXIPHOS complex (50). Considering that TOMM20 is an active player of mitochondrial metabolism and presumably responsible for the mitochondrial uptake of SPOCK1, closer scrutiny of their relationship may provide further clarification of the processes involved. Although our phosphokinase array revealed significant downregulation of Yes and other members of the Src family, its expression in HCC is still under debate (51, 52) without mechanistic data. According to a more recent report, inhibition of Yes by miR-210 decreases the proliferative capacity of hepatoma cells (53).

### 4.5 SPOCK1 Intracellular or Extracellular?

Although mRNA expression of SPOCK1 depends upon the experimental conditions when using hepatoma cell lines, the intracellular presence of the protein is not influenced either by silencing or transfection. This indicates that SPOCK1 is secreted to the culture media. Certainly, SPOCK1 concentration in the culture medium changes according to the actual experimental conditions; it is decreased after silencing and increased after SPOCK1 transfection, supporting the idea that SPOCK1 is a secreted proteoglycan (36). However, its synthesis was not restricted to stromal cells, as opposed to an earlier report (36). These findings are in direct contradiction with those we detected in human livers, where the cytoplasm of cirrhotic and transformed hepatocytes was loaded with considerable amounts of SPOCK1. This raises several questions: is the signal peptide injured? Are there other structural alterations of the proteoglycan? Do cancer cells need more intracellular SPOCK1 for their functions? Is the proteoglycan removed into the circulation? Further studies are needed to answer these questions.

### 4.6 Overexpression of SPOCK1 Coincides With Downregulation of Syndecan-1 in Human Hepatocellular Carcinoma

In experimental hepatocarcinogenesis, syndecan-1 overexpression provides protection against the development of cancer, and in addition to other factors, the downregulation of SPOCK1 could be observed throughout the experimental period (9). Since in human HCCs syndecan-1 lacks the protective effect, we assume that the increased syndecan-1 expression is a requirement for the protection against the harmful effects of SPOCK1. Further studies are necessary to understand those events that regulate the interaction of the two proteoglycans. Potential mechanisms can involve forced shedding of syndecan-1 and/or its decreased synthesis. The effect seems to be related to tumor aggressiveness because cells of well-differentiated tumors can rescue their cell surface syndecan-1. In contrast, no detectable amounts of the proteoglycan were found on the surface of micrometastases. Concerning other proteoglycans, no publications were found in PubMed about their interaction with SPOCK1.

Syndecan-1 is the most studied proteoglycan of the liver. Besides it functions as a lipoprotein receptor (54), it interferes with lipid metabolism by inhibiting the synthesis of FASN and modulating the Akt-mTOR and Wnt signaling, in this way protecting again NAFLD and experimental carcinogenesis (9). Its expression increases in liver fibrogenesis and liver cirrhosis (55), and its activity as the receptor of HCV is also confirmed (56).

Recently, syndecan-4 was also published to be a serum marker of NAFLD (7) and also acts as a receptor of HCV (57), not only infecting the hepatocytes but also responsible for the submission of the virus from men to men infecting anal Langerhans cells (58). Besides syndecan-1, syndecan-4 also regulates the Wnt signaling (59). By masking the functional domain of osteopontin, syndecan-4 inhibits the development of osteopontin-induced acute liver injury (60).

### 4.7 Possible Cooperation of Liver Proteoglycans in the Promotion of Cancer

In preexisting literature, only glypican-3 and agrin have been recognized as proteoglycans with oncogenic potential in the liver. Based on the data presented herein, SPOCK1 will likely join this company. Glypican-3 is a cell membrane-anchored proteoglycan (61), while agrin is present in the basement membranes of tumor vasculature, bile ducts, and the portal area (62). According to our results, SPOCK1 is present in the mitochondria of the HCC cells. Due to their distinct localization, a direct interaction between these proteoglycans is improbable; their effects may nevertheless combine. Glypican exerts its action *via* the Wnt pathway by upregulating β-catenin, facilitating the transcription of proteins with oncogenic potential while simultaneously downregulating Hedgehog signaling (63). Agrin regulates focal adhesion integrity, facilitates migration, and promotes EMT by signaling through the Musk receptor (64). Furthermore, it is involved in the downregulation of the Hypo pathway *via* transducing matrix stiffness by utilizing both Musk and integrin receptors (65). SPOCK1 can stimulate the mTOR pathway (38) and utilize Wnt signaling (28, 66). Additionally, the fact that it promotes resistance against third-generation TK inhibitors in lung cancer (39) indicates its involvement in EGFR signaling. The potential cooperation of glypican-3, agrin, and SPOCK1 in liver oncogenesis should be elucidated in future experimental models.

### 4.8 Conclusion

**Figure 14:**
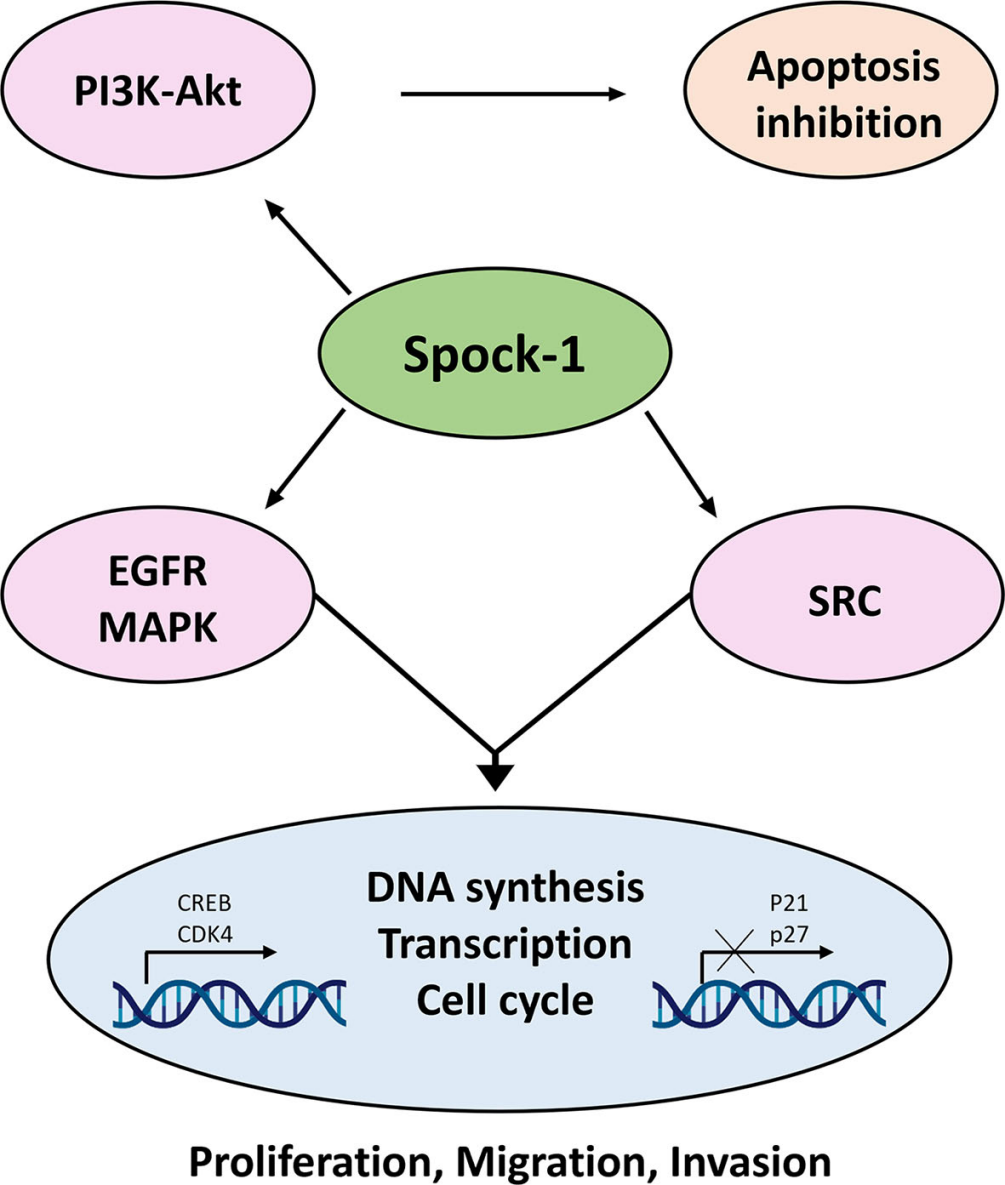
Action of SPOCK1 in hepatoma cell lines. In harmony with previous results, Spock-1 stimulates the Akt-mTOR pathway. Akt activation results in apoptosis inhibition. The mitochondrial localization of the proteoglycan may interfere in other ways as well with the apoptotic activity, which needs further evaluation. SPOCK1 silencing resulted in the downregulation of the EGFR tyrosine kinase receptor and members of the Sarc tyrosine kinase family, indicating the stimulatory potential of the proteoglycan on the signaling activity, partly through the MAPK pathway. Furthermore, SPOCK1 inhibition pointed to a decrease of two nuclear proteins CREB and CDK4; the former is a multivalent transcription factor with strong oncogenic potential, and the latter is the stimulator of cell cycle. Both nuclear proteins can be upregulated *via* MAPK signaling. Furthermore, CREB establishes interaction with several nuclear factors, facilitates DNA synthesis, and regulates chromatin function as well. Its action is in a good agreement with the effect of SPOCK1 on DNA synthesis, cell migration, and invasion (43).

Data collected in the last 12 years indicate that SPOCK1 is a proteoglycan with oncogenic potential. Its presence in tumor cells increases with tumor aggressiveness, and it can influence the function of receptor tyrosine kinases, intracellular phosphokinases, and DNA synthesis and alleviates the protective potential of syndecan-1 against HCC. Most likely, after secretion to the ECM, SPOCK1 establishes interaction with TGFβ1 and promotes EMT (67). SPOCK1 may additionally interact with other, currently unidentified, proteins and cellular mechanisms (**Figure 14**). Because in human and experimental hepatocellular carcinoma its stromal expression was hardly detected after secretion, SPOCK1 is likely to be cleared by the circulation. This finding may serve as a diagnostic tool in the not too distant future.

## Data Availability Statement

The original contributions presented in the study are included in the article/**Supplementary Material**. Further inquiries can be directed to the corresponding author.

## Ethics Statement

The studies involving human participants were reviewed and approved by Semmelweis University Regional and Institutional Committee of Science and Research Ethics (TUKEB permit number: 155/2012) and Medical Research Council Committee of Science and Research Ethics (permit number: 61303-2/2018/EKU). Written informed consent for participation was not required for this study in accordance with the national legislation and the institutional requirements. The animal study was reviewed and approved by the Ethics Committee of the Animal Health Care and Control Institute, Csongrád County (protocol code XVI/03047-2/2008).

## Author Contributions

Conceptualization: KB, ZS, LT, LV, and IK. Methodology: KK, BP, AR, and IK. Data curation: LV and IK. Formal analysis: LV, AR, KK, and IK. Funding acquisition: KB, AK, KD and IK. Investigation: LV, AK, KK, LT, GP, BP, and IK. Resources: AS and IK. Validation: KD, AS, KB, and IK. Writing: LV and IK. Writing—review and editing: KD, KB, and IK. Visualization: AR, GP, and IK. Supervision: ZS, AK, and IK. Project administration: IK. All authors listed have made a substantial, direct, and intellectual contribution to the work and approved it for publication.

## Funding

This work was supported by the Hungarian Scientific Research Fund (grants 67925, 100904 and 119283 to IK; FK 138673 to KD and FK 138593 to KB) and European Union Horizon 2020 Marie Skłodowska-Curie Actions (MSCA) Research and Innovation Staff Exchange Evaluations (RISE) project (grant 645756): “GLYCANC—Matrix glycans as multifunctional pathogenesis factors and therapeutic targets in cancer” (to IK and KB), EFOP-3.6.3-VEKOP-16-2017-00009 (to LV).

## Conflict of Interest

The authors declare that the research was conducted in the absence of any commercial or financial relationships that could be construed as a potential conflict of interest.

## Publisher’s Note

All claims expressed in this article are solely those of the authors and do not necessarily represent those of their affiliated organizations, or those of the publisher, the editors and the reviewers. Any product that may be evaluated in this article, or claim that may be made by its manufacturer, is not guaranteed or endorsed by the publisher.
