# Corrigendum: SPOCK1 promotes the development of hepatocellular carcinoma

**DOI:** 10.3389/fonc.2023.1203745

**Published:** 2023-04-19

**Authors:** Lóránd Váncza, Katalin Karászi, Bálint Péterfia, Lilla Turiák, Katalin Dezső, Anna Sebestyén, Andrea Reszegi, Gábor Petővári, András Kiss, Zsuzsanna Schaff, Kornélia Baghy, Ilona Kovalszky

**Affiliations:** ^1^ 1st Department of Pathology and Experimental Cancer Research, Semmelweis University, Budapest, Hungary; ^2^ Faculty of Information Technology and Bionics, Pázmány Péter Catholic University, Budapest, Hungary; ^3^ MS Proteomics Research Group, Research Centre for Natural Sciences, EötvösLoránd Research Network, Budapest, Hungary; ^4^ 2nd Department of Pathology, Semmelweis University, Budapest, Hungary

**Keywords:** hepatocellular cancer (HCC), SPOCK1 in the liver, SPOCK1 and syndecan-1, SPOCK1 in mitochondrion, effect of SPOCK1 inhibition and overexpression

In the published article, there was an error in the Funding statement. The Funding statement for the Hungarian Scientific Research Fund was displayed as “(grants 67925, 100904 and 119283 to IK and FK 138673 to KD)”. The correct Funding statement appears below.

This work was supported by the Hungarian Scientific Research Fund (grants 67925, 100904 and 119283 to IK; FK 138673 to KD and FK 138593 to KB) and European Union Horizon 2020 Marie Skłodowska-Curie Actions (MSCA) Research and Innovation Staff Exchange Evaluations (RISE) project (grant 645756): “GLYCANC—Matrix glycans as multifunctional pathogenesis factors and therapeutic targets in cancer” (to IK and KB), EFOP-3.6.3-VEKOP-16-2017-00009 (to LV).

The authors apologize for this error and state that this does not change the scientific conclusions of the article in any way. The original article has been updated.

