# Supplementary material for: SPOCK1 Promotes the Development of Hepatocellular Carcinoma
Source: Front Oncol. 2022 Feb 3;12:819883. doi: 10.3389/fonc.2022.819883 (PMC8853618; doi:10.3389/fonc.2022.819883)
Supplement: Supplementary file 1 [file DataSheet_1.pdf]

Supplementary Material

1 Supplementary Table

1.1 Supplementary Table 1. Antibodies used

Primary Antibody	Host species, isotype	Manufacturer	Cat. No.	Dilution			
				IHC	ICC	Blot	WES
Anti-BrdU	Mouse, monoclonal, (clone B44)	Becton, Dickinson and Company BD, Franklin Lakes, NJ USA	347580		1:50		
CDK4	Mouse, monoclonal, (clone DCS-31 + DCS-35)	Thermo Fisher Scientific, Fremont, CA, USA	MS616P0			1:1000	
CHD1L	Rabbit, polyclonal	Merck KGaA, Darmstadt, Germany	HPA028670	1:1000			
Cleaved Caspase-3	Rabbit, monoclonal (clone 5A1E)	Cell Signaling Technology, Danvers, MA, USA	9664			1:1000	
Cross-Adsorbed Secondary Antibody, Alexa Fluor 568	Goat anti-Rabbit IgG	Invitrogen, CA, USA	A11011		1:200		
GAPDH	Rabbit, monoclonal (clone 14C10)	Cell Signaling Technology, Danvers, MA, USA	2118				1:50

Highly Cross-Adsorbed Secondary Antibody, Alexa Fluor 488	Goat anti-Mouse IgG	Invitrogen, CA, USA	A11029		1:200		
p21	Rabbit, monoclonal (clone EPR18021)	Abcam, Cambridge, UK	ab188224			1:1000	
p27	Rabbit, polyclonal	Abcam, Cambridge, UK	ab64949			1:1000	
p53	Rabbit, monoclonal (clone EP155Y)	Abcam, Cambridge, UK	ab33889			1:1000	
pAKT (T308)	Rabbit, monoclonal (clone C31E5E)	Cell Signaling Technology, Danvers, MA, USA	2965			1:1000	
Spock1	Rabbit, polyclonal	Merck KGaA, Darmstadt, Germany	HPA07450	1:100	1:200	1:1000	1:50
Tomm20	Mouse, monoclonal (clone F-10)	Santa Cruz Biotechnology Inc., Dallas, TX, USA	sc-17764		1:50		
β-Actin	Mouse, monoclonal, (clone AC-74)	Merck KGaA, Darmstadt, Germany	A2228			1:2000	
β-Catenin	Rabbit, polyclonal	Cell Signaling Technology, Danvers, MA, USA	9562			1:1000	

2 Supplementary Figure

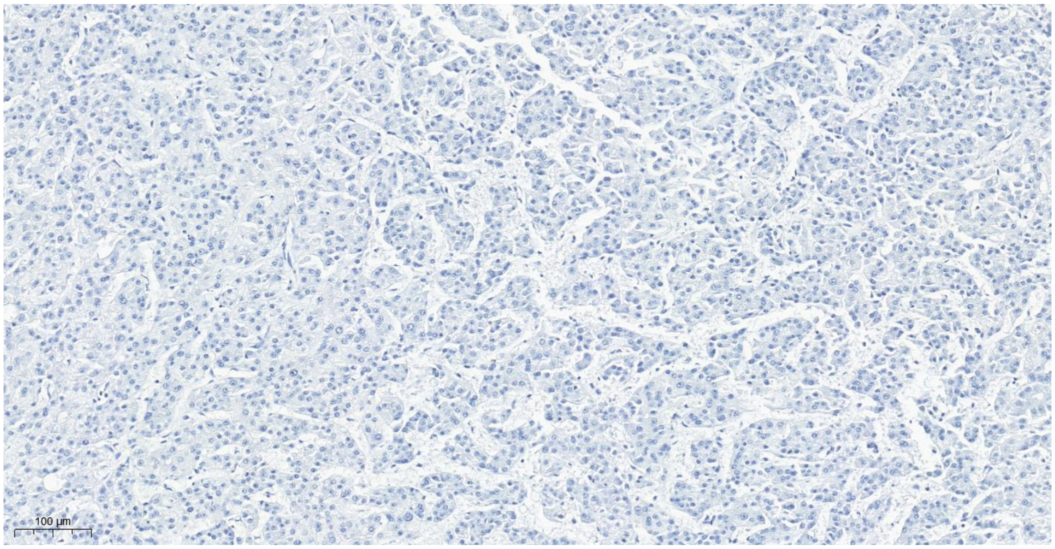

Supplementary Figure 1. Negative control of Figure 1. The Figure does not contain reactivity specific for SPOCK1.

3 Raw blots

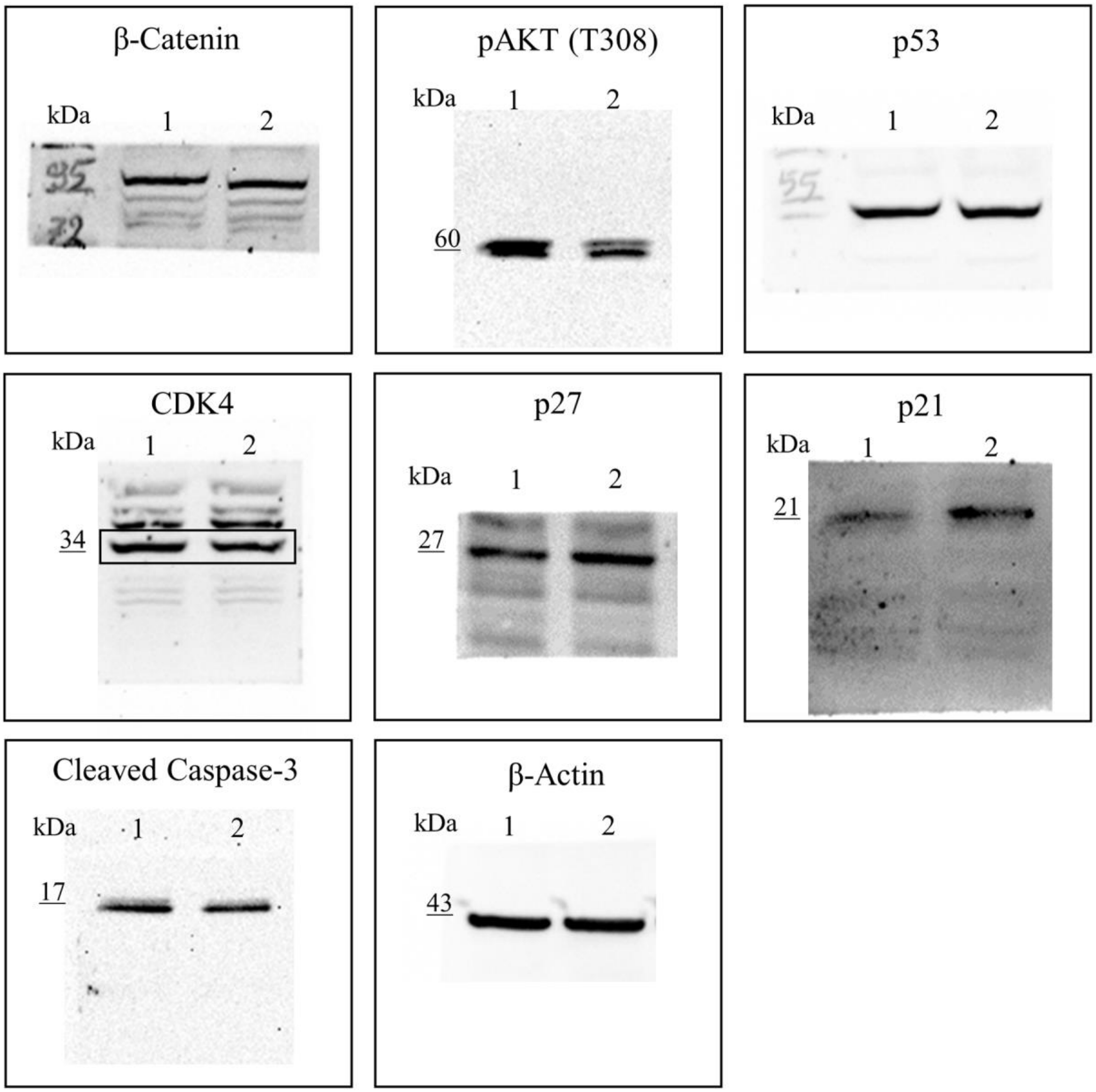

Supplementary Figure 2. Raw data of Figure 10. 1- HLE Control siRNA, 2-HLE SPOCK1 siRNA

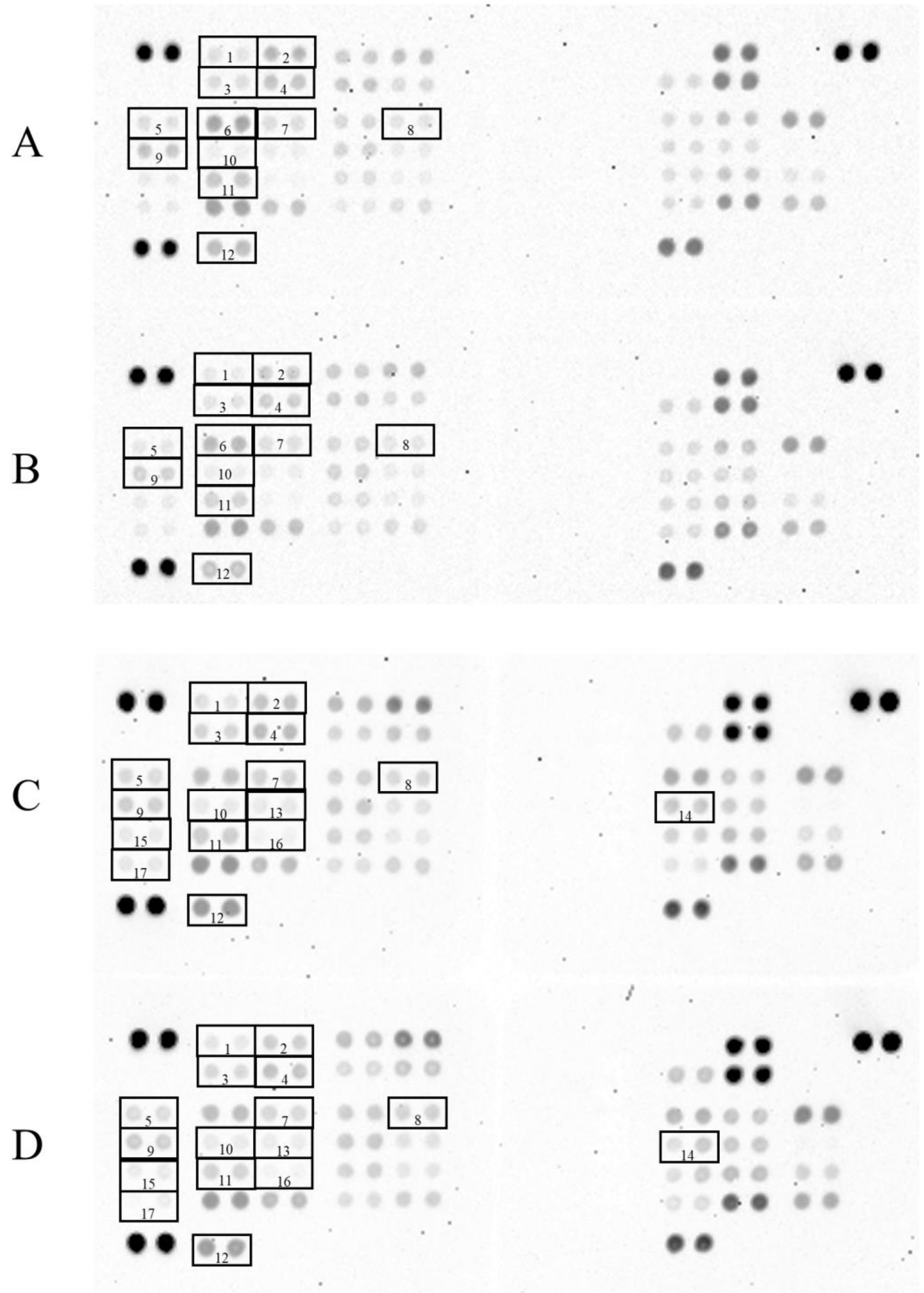

Supplementary Figure 3. Raw data of Figure 9. (A) HLE Control siRNA; **(B)** HLE SPOCK1 siRNA; **(C)** Huh7 Control siRNA; **(D)** Huh7 SPOCK1 siRNA; 1-p38α; 2-ERK1/2; 3-EGFR; 4-MSK1/2; 5-TOR; 6-CREB; 7-HSP27; 8-β-Catenin; 9-Src; 10-Lyn; 11-Yes; 12-PRAS40; 13-Lck; 14-p70S6K; 15-Fyn; 16-Fgr; 17-Hck.
